# Expansion of antibody reactivity in the cerebrospinal fluid of multiple sclerosis patients – follow-up and clinical implications

**DOI:** 10.1186/1743-8454-2-3

**Published:** 2005-06-27

**Authors:** Hela-Felicitas Petereit, Dirk Reske

**Affiliations:** 1 Department of Neurology, University of Cologne, Kerpener Str. 62, D-50924 Cologne, Germany

## Abstract

**Background:**

An intrathecal polyspecific antibody response is a well known finding in multiple sclerosis. However, little is known about the evolution of intrathecal antibodies over time and their impact on the disease progress. Therefore, we focused in this study on the intrathecal polyspecific antibody response in multiple sclerosis.

**Methods:**

Here we present a follow-up study of 70 patients with multiple sclerosis over 1 to 106 months. Serum and cerebrospinal fluid sample pairs were obtained from 1 to 5 consecutive lumbar punctures. CSF cell count, the IgG index, local IgG synthesis, oligoclonal bands and the antibody index for measles, rubella or varicella zoster were calculated. Results were analysed with regard to clinical characteristics of the patients.

**Results:**

Once an intrathecal antibody response was established, it persisted. De novo antibody response against measles virus developed in 7% of the patients between the first and the second spinal tap. In two of seven patients where 5 consecutive CSF samples were available, the intrathecal antibody response expanded from one to three antigens. Furthermore, an intrathecal measles antibody production was associated with a rapid progression of the disease.

**Conclusion:**

These data stress the importance of activated B cells for the disease process and the clinical outcome in multiple sclerosis.

## Background

An elevated immunoglobulin G (IgG) index and the presence of oligoclonal bands (OCB) in the cerebrospinal fluid (CSF) are a hallmark of multiple sclerosis (MS) [1,2]. Although this finding is not specific for MS, 72 % percent of patients present with an elevated IgG index and even 98 % show an oligoclonal distribution of IgG bands exclusively in the CSF [3,4]. Intrathecal IgG is thought to be the product of B lymphocytes residing in the brain of MS patients after they have crossed the blood brain barrier in an activated state with the help of various co-stimulatory signals [5]. Instead of undergoing apoptosis, the B cells expand clonally within the central nervous system (CNS) giving rise to a persistent antibody production [6]. Despite intense investigations, no single antigen against which the antibodies might be directed has been isolated so far. In contrast, the intrathecal antibody response covers a large number of CNS and non-CNS antigens as well as various pathogens [7-14], including the viral antigens such as measles, rubella and varicella zoster [15]. In up to 96 % of MS patients an intrathecal antibody production against at least one of the three antigens has been found [3,16]. Little is known, however, about the clinical significance of these findings. Previous studies attempting to evaluate the long-term evolution of intrathecal viral antibodies were hampered by technical shortcomings such as few sensitive detection methods and an absence of correction for blood-CSF-barrier disturbance [17,18]. Hence, the findings of these preliminary studies were partially contradictory with regard to the stability of the CSF antibody production [17,18]. Furthermore, no correlation to the clinical course could be demonstrated [17,18]. Here we report the results of a follow-up study on 70 MS patients from which at least two CSF analyses including cell count, IgG index, local IgG synthesis, antibody specific index and oligoclonal bands were available. The clinical implications of the immunological findings are discussed.

## Methods

### Patients

The study used 70 consecutive patients with definite MS according to the criteria of Poser [19] and a primary relapsing course. All patients had at least two spinal taps, mostly during disease exacerbations. Three lumbar punctures (LP) were performed in 26 patients, four in 12 patients and five in 7 patients. Patients were characterized clinically by age, sex, disease duration, course of the disease and the expanded disability status scale (EDSS), documented at the time of the first LP. Furthermore, the progression index was calculated as the ratio of the EDSS and the disease duration for each patient. Patients with corticosteroid treatment in the last four weeks or with immunomodulatory or immunosuppressive therapy in the last 3 months prior to the first LP were excluded. Cerebrospinal fluid and serum sample pairs were analyzed for cell count in the CSF, oligoclonal bands in serum and CSF, local IgG synthesis, IgG index and antibody index for the following antigens: measles, rubella and varicella zoster virus. We focused only on these specific antigens because of the frequent detection of these antigens within CSF in the case of MS described in prior studies [3].

### Cell count

The CSF cell count was determined immediately after LP. For this purpose, 90 μl of CSF were stained with 10 μl dye containing 20% crystal violet solution, 20 % glacial acetic acid and 60% H_2_O. Cells were enumerated in a Fuchs-Rosenthal counting chamber.

### IgG index

The intrathecal IgG production was quantitated by the IgG index. For this purpose, albumin and IgG were measured in matched serum and CSF pairs by nephelometry according to the manufacturer's instructions using commercially available kits (antiserum against human albumin or IgG, respectively) and the BN 100 nephelometer (both Dade Behring GmbH, Marburg, Germany). The IgG index was calculated according to the following formula: (CSF IgG × serum albumin) / (serum IgG × CSF albumin). An IgG index above 0.7 was indicative of an intrathecal IgG synthesis [20].

### Local IgG synthesis

We quantitated the local IgG synthesis within the CSF with the help of a method described by Reiber and colleagues [3]. Therefore, we used the following formula: Ig_loc _= [Q_Ig _- Q_lim _(Ig)] × Ig(serum) (Q = quotient CSF: serum). Details of the calculation have been described previously [21].

### Oligoclonal bands (OCB)

All samples were analyzed directly after performing the LP without freezing, to minimize post-sampling changes. The presence of oligoclonal bands in the CSF indicated an intrathecal IgG production. For detection of OCB, isoelectric focusing was performed on matched serum and CSF sample pairs. The serum and CSF samples were diluted to the same IgG concentration and run on polyacrylamide gel precoated with ampholytes (pH between 4.5 and 10.0) at increasing voltage according to the manufacturer's instructions (Servalyt Precotes, Serva Electrophoresis GmbH, Heidelberg, Germany). Subsequently, a silver staining with commercially available reagents was done as indicated by the manufacturer (Serva Electrophoresis GmbH). The patterns were interpreted qualitatively by comparing the presence or absence of OCB in the serum and CSF.

### Antibody specific index (AI)

During the whole study period, antibody production against measles, rubella and varicella zoster virus was analysed in matched serum and CSF pairs by commercially available, specific enzyme immune assays (Enzygnost by Dade Behring) on an ELISA II processor using the alpha-method (Dade Behring). The AI was calculated according to the following formula: AI = Q spec. / Q lim. with Q spec. = CSF antibodies / serum antibodies and Q lim. being calculated from the CSF / serum albumin ratio according to the Reiber formula [21]. AI larger than 1.4 indicate an intrathecal antibody synthesis against the given antigen.

### Statistical analyses

This study was carried out as retrospective case-control study. The mean values of cell count, IgG index, local IgG synthesis and AI were compared for the whole group at the different time points (LP 1 to 5) by non-parametric tests for dependent samples (Friedman test). The percentage of patients positive for OCB or with a positive AI against measles at the 1^st ^and 2^nd ^LP, respectively, was compared with the Fisher's Exact test.

Different clinical subgroups, i.e. those with a high or low EDSS, were analyzed for their mean measles AI with the help of a median split. The mean measles AI in the subgroups was compared with a non-parametric test for independent samples (Mann Whitney U test).

## Results

### Patient characteristics

A total of 70 patients, 45 women and 25 men, were included in this study. The mean age of the patients was 38 years (range 21 to 67 years). All patients had a primary relapsing course of the disease. The mean disease duration was 4.5 years with a range of one to 37 years. The mean EDSS at study entry was 2.9 (standard deviation SD 1.33). The mean time from the first LP to the second was 15 months with a range from 1 to 81 months. The mean time from the first to the last LP was 22 months (range: 1 to 106 months) in all patients. In 30 patients the range between the first and second LP was at least 12 months. In 24 patients the time between these LPs was less than 6 months. At the occasion of the second LP, 22 patients received an immunomodulatory or immunosuppressive treatment (interferon beta, glatiramer acetate, intravenous immunoglobulin, azathioprine). It has to be stressed that the study population is inhomogeneous. If the LPs were sorted by the follow-up years in which they had been arranged, no new information could be observed. This might be due to the wide range of time between the different LPs in this patient cohort (1 to 106 months). The data are not shown.

### Mean IgG index, local IgG synthesis and AI

There was no significant change of the mean IgG index, local IgG synthesis or AI for any of the viral antigens investigated over time (p = 0.414 for IgG index, p = 0.578 for local IgG synthesis, p = 0.673 for measles AI, p = 0.984 for rubella AI, p = 0.941 for varicella zoster AI, Friedman test). The data are summarized in Table 1. In addition, there was no significant change in the mean cell count (p = 0.291, Friedman test, data not shown).

**Table 1:** Mean and SD of IgG index, local IgG synthesis (Ig_loc_) and antibody index (AI) for measles, rubella and varicella zoster virus at each successive lumbar puncture (LP). The number of patients in each category of serial LP is given. All included patients received at least two serial LPs (first and second). Additional LPs were only obtained in the mentioned number of patients.

LP no.	No. of patients with the respective LP	IgG index	Ig_loc _(mg/L)	AI measles	AI rubella	AI varicella zoster
1.	70	1.3 ± 0.75	33 ± 5,0	3.4 ± 3.29	4.6 ± 7.11	3.6 ± 5.86
2.	70	1.3 ± 1.58	25 ± 4,1	3.5 ± 3.67	4.8 ± 7.67	3.6 ± 5.85
3.	26	1.4 ± 0.99	46 ± 1,1	3.9 ± 5.59	5.4 ± 9.28	4.7 ± 7.28
4.	12	1.2 ± 0.73	28 ± 0,9	2.8 ± 2.09	6.7 ± 14.78	4.4 ± 8.74
5.	7	1.0 ± 0.39	19 ± 4,8	2.1 ± 0.89	1.9 ± 1.28	1.9 ± 1.46

### OCB and AI positive patients

**Figure 1:**
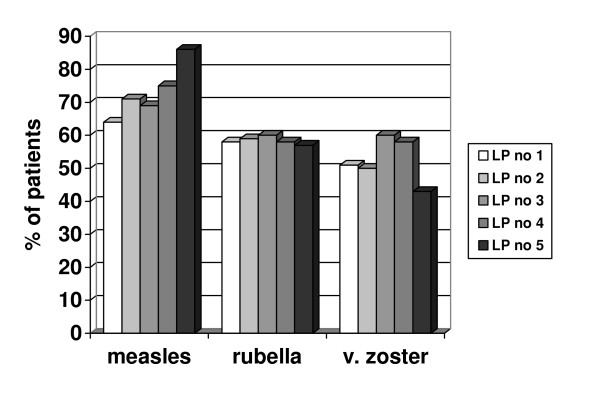
Percentage of patients with an intrathecal antibody synthesis against measles, rubella, and varicella zoster virus at five successive spinal taps (LP, n = 70 for LP 1 and 2, n = 26 for LP 3, n = 12 for LP4 and n = 7 for LP5). There was a significant increase (*p *<0.05) in patients positive for antibody synthesis against measles virus over time. There were no significant changes in rubella or varicella zoster antibody synthesis.

As expected, the presence of OCB in the CSF was more sensitive in the detection of an intrathecal IgG production than a positive IgG index [22,23]. For instance, at the 1^st ^LP 94% of patients had positive oligoclonal CSF bands whereas 88% of patients had a positive IgG index. Once positive the OCB bands remained positive during the follow-up. The percentage of patients who were positive for oligoclonal bands in the CSF increased after the 1^st ^LP from 94 to 100 %. Likewise, the relative number of patients with an intrathecal antibody synthesis against measles virus increased significantly over time (Figure 1). No obvious change in rubella and varicella zoster antibody positive patients over time was observed. This significant increase in patients with an intrathecal antibody production against measles was seen in both patient subgroups irrespective of whether or not they were treated with any immunomodulatory treatment mentioned above at the 2^nd ^LP or not (Table 2).

**Table 2:** Percentage of patients who had oligoclonal bands in the CSF or a positive AI for measles at the respective lumbar puncture (LP).

		1^st ^LP	2^nd ^LP	p value
Untreated patients	OCB	96	100	not done
	measles AI	68	73	0.013
Treated patients	OCB	91	100	not done
	Measles AI	55	68	0.001
All patients	OCB	94	100	not done
	Measles AI	64	71	<0.001

### Individual long-term follow-up studies

In 7 patients, data from 5 consecutive spinal taps were available. Except for patient no. 57, all data were derived from patients with a secondary progressive course of the disease and collected over a period of 1 to 6 years. Although the AI for the various antigens was not identical, 4 of the patients were stable over time with regard to their status as antibody producers (patients no. 16, 21, 41, 52). Of these patients, one was positive for measles alone, one for measles and rubella, one for all three viral antigens and one patient was antibody negative. The introduction of an immunomodulatory or immunosuppressive treatment at the 2^nd ^LP (interferon beta patients 16, 21 and 41, azathioprine patient no. 52) had no impact on the antibody production. Two additional patients experienced an expansion of their antibody response from one (rubella virus in both cases) to three antigens, despite treatment initiated at the 2^nd ^LP (interferon beta in patient no. 5 and azathioprin in patient no. 34).

### Correlation with clinical features

There was no significant difference in the mean AI against measles in patients with high or low disability (p = 0.995, Mann Whitney U test) at the 1^st ^LP. High disability was defined as an EDSS score above the median of 3.0. Furthermore, there was no difference in the mean measles AI in patients with long and short disease duration (median 2 years, p = 0.304). The EDSS scale and disease duration was used to calculate the progression index for each patient. A progression index of more than 1 (median), indicating an increase of at least 1 point on the EDSS scale per year was regarded as high. The mean AI against measles was significantly higher in patients with a high progression index than in those with a low progression index (p = 0.038). In the subgroup with a high progression index the mean measles AI was 4.2, whereas in the low progression index group a mean measles AI of 2.6 was measured. In the low progression index subgroup 53% of patients had an intrathecal antibody production against measles, but almost 74% were positive for measles in the high progression index subgroup. There were no significant differences in the local IgG synthesis when comparing the groups with high or low disability at the 1^st ^LP or the groups with a high or low progression index (p = 0.71, p = 0.962, respectively). In these respective groups, the IgG index was not changed (p = 0.165 high disability group; p = 0.828 high progression index).

## Discussion

There are three main findings of our study: i) Once an intrathecal antibody production is established it is maintained over time; ii) For the first time we provide evidence for an expansion of antibody reactivity in the CSF of MS patients; and iii) disease progression and B cell activation may be linked.

### Persistent IgG production

In our study patients positive for oligoclonal IgG bands in the CSF remained positive at the follow-up. These findings are in accordance with previous data indicating a clonally stable IgG production in the CSF over long periods [24]. This was true despite the introduction of immunomodulatory or immunosuppressive treatment. In 132 MS patients serial CSF analysis before and after 2 years of interferon beta treatment or placebo failed to reveal differences in the IgG index or OCB in either treatment group [25]. Even after autologous hematopoietic stem cell transplantation identical OCB compared to pre-treatment CSF analysis were found [26,27]. Similarly in our study, there was no effect of treatment on IgG production as documented by the IgG index or on the local IgG synthesis in the CSF. In addition to the well-known finding of a stable OCB production, we demonstrated for the first time that the intrathecal antibody production is stable over time as well. This means, once an intrathecal antibody response against measles, rubella or varizella zoster virus was established it persisted at the follow-up analyses for at least 4 years.

Although persistent IgG and antibody production is a well-known phenomenon in multiple sclerosis, the mechanisms remain unknown. It has been demonstrated recently in an experimental model of B cell migration over the blood brain barrier that activated human B cells readily cross the blood brain barrier with the help of adhesion molecules and chemokines [5]. Longevity of B cells and plasma cells is a common feature [28], and the microenvironment of MS lesions promotes the persistence and activation of B cells [29]. Although repeated antigenic stimulation favors longevity of B cells, persistance of antigen is not required for the survival of B cells [30]. Instead, high expression of anti-apoptotic proteins may be involved in the survival of B cells. Interestingly, increased expression of the apoptosis-inhibitory proteins Bcl-2 and FLIP were detected in B cells of MS patients [6]. Alltogether, microenvironment and antiapoptotic signaling might explain in part the persisting IgG and antibody production in the CSF of MS patients even in the absence of persistent antigen exposure.

### Expansion of antigen response

More intriguing was the finding that the number of patients positive for intrathecal OCB increased over time from 94 to 100%. It has been assumed that in early disease activated B cells might not yet have become resident in the CNS. Progression from a monoclonal to an oligoclonal CSF pattern has been reported in multiple sclerosis patients [31]. An increase in CNS clones after the initial stage of the disease has been suggested as a possible explanation. Further evidence for an expanding B cell activity during the course of the disease comes from a CSF analysis in which the prevalence of mature plasma cells was found to be higher in patients with longer disease duration [32].

In our study, an increasing number of patients showed a newly established intrathecal antibody response against measles virus: whereas 64% of patients were positive for measles at the 1^st ^LP, 71% became positive at the 2^nd ^and 86% at the 5^th ^LP. Furthermore, in two out of seven patients with repeated CSF analysis, there was an expansion of the B cell response from one to three viral antigens by the 5^th ^LP. To our knowledge, this is the first follow-up report on intrathecal polyspecific antibody production in multiple sclerosis patients. This follow-up for the first time provides evidence for an expansion of antibody reactivity over time in multiple sclerosis patients. However, this expansion of antibody is in contrast to the stable local IgG synthesis over time.

One possible explanation for a polyspecific intrathecal antibody production might be a bystander activation of B cells with a given antibody reactivity. This is possible in the presence or absence of T cells. If certain cytokines -namely tumor necrosis factor alpha- is present, IgG secretion by activated B cells may occur even in the absence of T cells [33]. Why preferentially measles, rubella, varicella zoster and to a lesser extent other virus reactive B cells become activated during the disease process remains unclear.

### Association between B cell activity and clinical aspects of MS

Although the evidence of B cell activity in the CNS is a common feature in MS, its clinical significance remains to be elucidated. Early studies on fluctuations in the CSF antibody production failed to show an association between antibody titers and the clinical course, possibly due to methodological shortcomings [17,18]. In contrast, a recent publication revealed an association between a very high IgG index and a rapidly progressing course of the disease [34]. Further evidence for an impact of B cell products on disease severity comes from a publication by Villar and colleague. They found an intrathecal IgM synthesis to be associated with a worse clinical outcome [35]. Accordingly, a predominance of B cells over T cells and macrophages in the CSF has been shown to be associated with a more rapid disease progression, but not with a longer disease duration or a higher disability score [36]. Interestingly, when our patients were divided into two subgroups according to the rate of disease progression, there were more measles positive patients in the rapidly progressive subgroup. Furthermore, the AI for measles was significantly higher in those with a rapid progression of disability.

## Conclusion

In summary, our data indicate that once an intrathecal antibody production has been established it is stable over time. Furthermore, an expansion in antibody specifity occurred in a proportion of patients. The presented data support the hypothesis that a strong B cell activation is associated with a worse clinical outcome in MS.

## List of abbreviations

AI – antibody specific index

CNS – central nervous system

CSF – cerebrospinal fluid

EDSS – expanded disability status scale

Ig – immunoglobulin

LP – lumar pucture

MS – multiple sclerosis

OCB – oligoclonal bands

## Competing interests

The author(s) declare that they have no competing interests.

## Authors' contributions

H-FP gave the idea of this study and participated in the design of the study. The statistical analysis and the design of the study was performed by DR. Both authors read and approved the final manuscript.
